# The effects of resuscitation with different plasma products on endothelial permeability and organ injury in a rat pneumosepsis model

**DOI:** 10.1186/s40635-023-00549-9

**Published:** 2023-09-20

**Authors:** Daan P. van den Brink, Derek J. B. Kleinveld, Annabel Bongers, Jaël Vos, Joris J. T. H. Roelofs, Nina C. Weber, Jaap D. van Buul, Nicole P. Juffermans

**Affiliations:** 1 Department of Intensive Care Medicine, Amsterdam UMC, University of Amsterdam, Meibergdreef 9, 1105 AZ Amsterdam, The Netherlands; 2 Laboratory of Experimental Intensive Care and Anesthesiology, Amsterdam UMC, University of Amsterdam, Amsterdam, The Netherlands; 3 Department of Intensive Care Medicine, Erasmus MC, Erasmus University of Rotterdam, Rotterdam, The Netherlands; 4 Department of Pathology, Amsterdam UMC, University of Amsterdam, Amsterdam, The Netherlands; 5 Cardiovascular Sciences, Amsterdam UMC, Amsterdam, The Netherlands; 6 Molecular Cell Biology Lab at Department Molecular Hematology, Sanquin Research and Landsteiner Laboratory, Amsterdam, The Netherlands; 7 Leeuwenhoek Centre for Advanced Microscopy (LCAM), Section Molecular Cytology at Swammerdam Institute for Life Sciences (SILS) at University of Amsterdam, Amsterdam, The Netherlands; 8 Department of Intensive Care Medicine, OLVG Hospital, Amsterdam, The Netherlands

**Keywords:** Albumin, Endothelial dysfunction, Plasma, Resuscitation, Sepsis, Shock, Transfusion

## Abstract

**Background:**

Endothelial injury and permeability are a hallmark of sepsis. Initial resuscitation of septic patients with crystalloids is associated with aggravation of endothelial permeability, which may be related either to low protein content or to volume. We investigated whether initial resuscitation with different types of plasma or albumin decreases endothelial dysfunction and organ injury in a pneumosepsis rat model compared to the same volume of crystalloids.

**Study design and methods:**

Sprague–Dawley rats were intratracheally inoculated with *Streptococcus pneumoniae.* Twenty-four hours after inoculation, animals were randomized to 2 control groups and 5 intervention groups (*n* = 11 per group) to receive resuscitation with a fixed volume (8 mL/kg for 1 h) of either Ringer’s Lactate, 5% human albumin, fresh frozen plasma derived from syngeneic donor rats (rFFP), human-derived plasma (hFFP) or human-derived solvent detergent plasma (SDP). Controls were non-resuscitated (*n* = 11) and healthy animals. Animals were sacrificed 5 h after start of resuscitation (*T* = 5). Pulmonary FITC-dextran leakage as a reflection of endothelial permeability was used as the primary outcome.

**Results:**

Inoculation with *S. Pneumoniae* resulted in sepsis, increased median lactate levels (1.6–2.8 mM, *p* < 0.01), pulmonary FITC-dextran leakage (52–134 µg mL^−1^, *p* < 0.05) and lung injury scores (0.7–6.9, *p* < 0.001) compared to healthy controls. Compared to animals receiving no resuscitation, animals resuscitated with rFFP had reduced pulmonary FITC leakage (134 vs 58 µg/mL, *p* = 0.011). However, there were no differences in any other markers of organ or endothelial injury. Resuscitation using different human plasma products or 5% albumin showed no differences in any outcome.

**Conclusions:**

Resuscitation with plasma did not reduce endothelial and organ injury when compared to an equal resuscitation volume of crystalloids. Rat-derived FFP may decrease pulmonary leakage induced by shock.

**Supplementary Information:**

The online version contains supplementary material available at 10.1186/s40635-023-00549-9.

## Background

A hallmark of sepsis is the presence of a dysregulated inflammatory host immune response with ensuing endothelial injury and hyperpermeability [1–3]. Under healthy conditions, the inner endothelial cell wall is lined with a matrix of proteoglycans and glycoproteins, termed the glycocalyx [4]. This structure is integral in regulating vascular permeability, facilitating leukocyte migration [5] and maintaining coagulation homeostasis [6]. During sepsis, constituents of the glycocalyx such as syndecan-1 and thrombomodulin are shed into the circulation, which is associated with worse patient outcomes [2, 7].

The primary initial treatment for patients with septic shock consists of fluid resuscitation to ensure adequate tissue perfusion [8]. However, low protein-content fluids, such as crystalloids, may harm the endothelial barrier and glycocalyx, as shown *in vitro* [9, 10]. In septic patients, fluid resuscitation with crystalloids is dose-dependently associated with an increase in circulating levels of constituents of the glycocalyx [11], although it is not clear whether this association is merely reflecting disease severity. High protein-content fluids, such as albumin and plasma, may be less harmful [12]. Albumin 4%, when compared to crystalloids, reduced endothelial dysfunction, inflammation and oxidative stress in a rat endotoxemia model [13]. Moreover, the use of albumin as an add-on fluid therapy in sepsis may confer benefit in those with baseline hypo-albuminemia [14], although results of clinical trials on albumin indicated no benefit on mortality [15]. Plasma might be even more promising [12, 16]. In an experimental sepsis model, plasma reduced mortality, lung edema and syndecan-1 shedding compared to crystalloids [17]. Observational clinical data suggests that plasma is associated with reduced markers of glycocalyx degradation in septic shock [18]. In addition, as solvent detergent plasma (SDP) is depleted from cellular debris, this plasma product may confer a superior benefit on the endothelium than fresh frozen plasma (FFP), as suggested in a trial in patients undergoing aortic surgery and in observations in critically ill pediatric patients [19, 20].

Alternatively to protein content, the observed benefit of plasma may be related to the use of less volume, as plasma has a 3–4 times higher volume expansion capacity than crystalloids. The preclinical shock models comparing plasma to crystalloids have used significantly higher volumes of crystalloids [17, 21, 22]. As no studies have evaluated the effects of the same volume of fluids on endothelial and organ injury in sepsis, it is unclear whether benefit is due to the type of fluid or the difference in infused volume.

The primary aim of this study was to investigate the effects of initial resuscitation with a similar fixed-volume of crystalloids, and rat-derived fresh frozen plasma (rFFP) on shedding of glycocalyx constituents, endothelial permeability and organ injury in a rat pneumosepsis model. We hypothesized that resuscitation with rFFP, would limit pulmonary endothelial permeability, reducing organ injury compared to crystalloids. In addition, we investigated the effects of different human-derived protein-rich plasma products, including human-derived fresh frozen plasma (hFFP), human-derived solvent detergent plasma (SDP) and 5% human albumin on endothelial and organ injury. We hypothesized that SDP would be superior to FFP or albumin.

## Methods

### General information

This study was approved by the Animal Care and Use Committee of the Amsterdam University Medical Centers, location AMC at the University of Amsterdam, the Netherlands. The procedures were performed in accordance with the European Parliament directive (2010/63/EU) and the national law of the Experiments on Animals Act (Wod, 2014). 72 male Sprague Dawley rats (Envigo, Indianapolis, IN, USA), 12–13 weeks of age and weighing around 400 g, were group housed in standard cages under normal conditions of a 12:12-h light:dark cycle receiving food and water ad libitum for at least 7 days before the experiment. Studies were conducted and reported following the ARRIVE guidelines (Additional file 2: Appendix S2) [23].

### Pneumosepsis model

*Streptococcus pneumoniae* (serotype 3, ATCC 6303; Rockville, MD, USA) were cultured on Colombian Agar with 5% sheep blood plates (COS) (Biomerieux Benelux B.V, Zaltbommel, the Netherlands). After 12 h of growth, 5–6 separate colonies were isolated and transferred to 20 mL brain heart infusion (BHI) broth. After overnight incubation at 37 °C, the culture was centrifuged for 10 min at 2700 g, 20 °C (5804 R, Eppendorf AG, Hamburg, Germany). Afterwards, medium was removed and bacteria were washed with sterile 0.9% saline and centrifuged a second time for 10 min at 2700 g, 20 °C (5804 R, Eppendorf AG, Hamburg, Germany). Using a spectrophotometer (Secoman S250), optical density (OD) was measured. Assuming that an OD_620_ of 0.350 = 1 × 10^8^ colony forming units (CFU), inoculum was made using sterile 0.9% NaCl. Prepared inocula were plated on COS plates in serial tenfold dilutions and grown overnight to quantify.

Following anesthesia using 5% isoflurane/60% FiO_2_ mix, rats underwent intratracheal inoculation with 3–5 × 10^8^ CFU *S. Pneumoniae* (ATCC6303) (BSL-2) in 100 µl sterile 0.9% NaCl. In pilot experiments, this dose range induced pneumosepsis, defined as lactatemia with growth of S. *pneumonia* in lung and blood cultures, in all animals. Then, animals were transferred to their cages and allowed to wake up (Additional file 1: Fig. S1). During the experiments the following exclusion criteria were used: animals receiving too low (< 3 × 10^8^ CFU) or high (> 5 × 10^8^ CFU) amounts of S. *pneumoniae*. Animals without growth of S. *pneumonia* in either lung or blood cultures. Animals that died before start of intervention. Exclusion criteria were established before start of experiments.

Twenty-four hours after inoculation (*T* = 0), animals were randomized to 5 intervention and 2 control groups (*N* = 11 per group) with opaque envelopes using a 1:1 allocation. Intervention groups received resuscitation with either Ringer’s lactate (RL), 5% human albumin, rat-derived fresh frozen plasma (rFFP), human-derived fresh frozen plasma (hFFP) or human-derived solvent detergent plasma (SDP). Positive controls (*n* = 11) underwent all procedures while not receiving any resuscitation. Healthy controls (*n* = 6) underwent all procedures but were sham inoculated with 100 µL sterile NaCl 0.9%. Researchers were not blinded to group allocation during conduct of the experiment and data analyses. A 21G venflon was inserted in the tail vein under anaesthesia with 1–3% isoflurane/60% FiO_2_ while breathing spontaneously. After obtaining a baseline blood sample (*T* = 0). Fluids were administered at 8 ml/kg in 1 h (perfusor® fm; B Braun, West Bloomfield Township, MI, USA). Animals received no additional fluids between inoculation and start of resuscitation (*T* = 0).

After resuscitation, the catheter was removed and anesthesia was stopped. After 5 h, rats were exsanguinated through heart puncture (*T* = 5). Fifteen min before sacrifice, 0.5 mL saline containing 12.5 mg/ml fluorescein isothiocyanate (FITC)-labelled 70 kDa dextran (Sigma Aldrich, St. Louis, MO, USA) was administered through a newly placed tail vein catheter. Immediately after exsanguination, 2 mL of heparinized saline (1250 iE/mL, Leo Pharma BV, Amsterdam) was administered to prevent clotting. Thereafter, laparotomy and thoracotomy were performed, urine was collected by bladder puncture, and the inferior vena cava was cut. The circulation was flushed using 100 ml 0.9% NaCl, administered into the right ventricle, to remove excess intravascular FITC-dextran. Before flushing, the left pulmonary and renal hilum were ligated for wet-to-dry (W/D) ratios. After flushing, both lungs and kidneys were taken out for later assessment.

### Animal welfare

Animals were monitored during the whole study period using a modified M-CASS scoring sheet (Additional file 1: Results S2) [17, 24]. Scoring 4 points in any of the categories was considered a humane endpoint and would result in early termination by heart puncture under anesthesia. No analgesics were administered during the study period. Animals were monitored during predefined moments by the responsible researcher (After inoculation, T12, T24, T26.5 and T29) or animal caretaker (T8, T20). Monitoring frequency was increased when unusual progression of symptoms was witnessed. Autopsy was performed on all animals not reaching the end of the study period to assess cause of death.

### Intervention products

Rat fresh frozen plasma (rFFP) transfusion product was prepared from donor rats according to national blood bank standards. In short; rats were anesthetized using 5% isoflurane and whole blood was obtained through cardiac puncture using a 19G syringe containing 10% citrate–phosphate–dextrose (Sigma, USA). Blood was centrifuged at 2000G, 18 °C for 10 min after which plasma was separated centrifuged again at 2000G, 4 C° for 15 min (5804 R, Eppendorf AG, Hamburg, Germany). Thereafter, upper 2/3rd of plasma was taken, snap-frozen in liquid nitrogen and stored at − 80 °C. Human albumin (Albuman 200®, Prothya Biosolutions Netherlands, the Netherlands) was diluted 1:3 using sterile ringer’s lactate to a concentration of 5% and stored at – 80 °C. Both FFP (Sanquin, the Netherlands) and SDP (Omniplasma®, Octapharma, Switzerland) products were AB- and retrieved from Sanquin, the national blood bank. Prior to the experiment, all plasma products were aliquoted, snap frozen and stored at – 80 °C. At the day of the experiment, required infusion products were quickly thawed in a water bath at 37 °C, after which they were kept at room temperature until infusion.

### Measurements

Blood was drawn before resuscitation (*T* = 0) and before exsanguination (*T* = 5) (Additional file 1: Fig. S1). Blood gas analyses (RAPIDPoint 500, Siemens, Germany) and total blood counts (cell Coulter AC•T diff2 Hematology analyser, Beckmann, Germany) were performed at both timepoints. All other biochemical assessments were measured only at termination (*T* = 5). Blood was centrifuged twice (2000g, 15 min, acceleration 9, brake 0, 18 C˚; 5804 R, Eppendorf AG, Hamburg, Germany), aliquoted, snap frozen in liquid nitrogen and stored at – 80 °C till further evaluation.

### Pulmonary vascular leakage

To quantify the amount of pulmonary FITC-dextran leakage, the superior lobe of the right lung was thawed and homogenized using a radio immunoprecipitation assay (RIPA) buffer and tissue homogenizer (TH-115, Omni international, Kennesaw, Georgia, USA). 100 µL of homogenates were measured for fluorescence using a spectrophotometer (spectramax M2, molecular devices, San Jose, California, USA).

### Pulmonary histopathology

The left inferior lobe of the right lung was fixed in 10% formaldehyde and embedded in paraffin, after which 4-µm-thick sections were cut and stained with haematoxylin and eosin (H&E). A pathologist blinded for treatment allocation evaluated the lung tissue for the presence of oedema, bronchitis, endothelialitis, interstitial inflammation and thrombi formation on a scale of 0–4 (0 = absent, 1 = mild, 2 = moderate, 3 = severe, 4 = very severe), as described previously [25, 26]. For the full scoring list, see the supplement (Additional file 1: Table S1). No other organs were assessed for histopathology.

### Bacterial growth

The right inferior lobe of the lung was homogenized in sterile 0.9% NaCl. Lung homogenates were plated on COS plates in serial tenfold dilutions. Whole blood retrieved during termination (*T* = 5) was plated on COS plates, growth of *S.* *pneumonia* in whole blood was defined as bacteremia.

### Parameters of systemic and organ injury

As a reflection of systemic endothelial injury and glycocalyx degradation, soluble levels of syndecan-1 (Elabscience, USA), thrombomodulin (TM) (Lifespan Biosciences, Inc. USA), von Willebrand factor (vWF) (Elabscience, USA) and intercellular adhesion molecule-1 (ICAM-1) (Elabscience, USA) were measured using commercially available ELISA kits according to manufacturer guidelines. ELISA values that were lower than the reference value were set at the lowest detection range. To assess liver and kidney injury, aspartate transaminase (AST), alanine transaminase (ALT), albumin and creatinine were measured by standard enzymatic reactions using spectrophotometric, colorimetric or turbidimetric measurement methods.

### Sample size and statistical analyses

The primary outcome was pulmonary leakage of FITC-dextran. In a previous sepsis model, resuscitation with plasma compared to normal saline reduced syndecan-1 levels (21.8 vs 31.0 ng/mL) [17]. These data were extrapolated to a sample size with 7 groups. Using a one-way ANOVA analysis of variance (*V* = 17.5) and a common standard deviation of 9, the use of 10 rats had a power of 80% to reach a statistically significant difference in vascular leakage. With an expected mortality of 5% in our model, 11 rats were included per group.

Statistical analysis was done using SPSS Statistics V.26 (IBM). Graphs were made using GraphPad Prism 8 (GraphPad Software). All data were regarded as non-parametric and are shown as median with interquartile range (IQR). Data were tested for differences using the Kruskal Wallis test with post-hoc multiple comparisons. A *p* value < 0.05 was considered statistically significant. No correction was used for multiple testing. All data collected till the moment of death was used in analyses.

## Results

### Pneumosepsis model

**Fig. 1:**
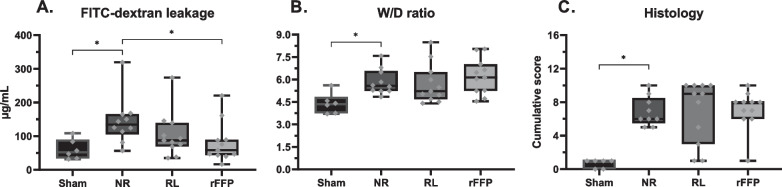
**Markers of pulmonary injury post resuscitation.** Data are presented as boxplots with median, interquartile ranges and minimum and maximum values showing all individual data points. *p < 0.05. *FITC* Fluorescein isothiocyanate, *NR* no resuscitation, *rFFP* rat fresh frozen plasma, *RL* Ringers Lactate, *W/D* Wet-to-dry

**Fig. 2:**
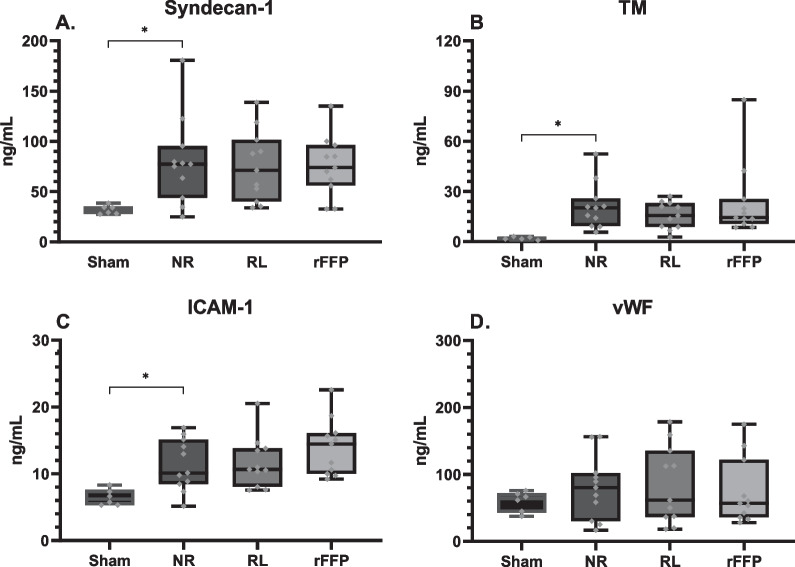
**Markers of endothelial injury post-resuscitation.** Data are presented as boxplots with median, interquartile ranges and minimum and maximum values showing all individual data points. *p < 0.05. *ICAM-1* intercellular adhesion molecule 1, *NR* no resuscitation, *rFFP* rat fresh frozen plasma, *RL* Ringers Lactate, *TM* Thrombomodulin, *vWF* von Willebrand factor

All animals inoculated with *S. pneumonia* (N = 66) developed pneumosepsis with growth of *S. pneumoniae* in both lung homogenate and blood cultures and lactatemia when compared to sham inoculated animals (*N* = 6) (median: 1.56 [IQR: 0.87–1.69] vs 2.77 [1.96–3.51] mM, p ≤ 0.01, Table 2). Septic rats had higher weight loss over 24 h compared to healthy control animals (8.1 [7.1–9.1] vs 0.9 [0.3–1.4] %, p ≤ 0.001). Septic animals had increased levels of potassium and base excess, whereas calcium levels and leucocyte and platelet counts dropped (Table 1). Moreover, septic animals had increased pulmonary oedema (higher W/D ratio) and higher pulmonary histology injury scores (Fig. 1), and elevated systemic parameters of endothelial injury when compared to healthy controls (Figs. 1, 2). Septic animals did not develop hepatic or renal injury when compared to healthy control animals as levels of ALT, AST, albumin and creatinine were comparable between all groups (Table 2).

**Table 1:** Pre-resuscitation parameters (*T* = 0)

Parameter	Sham (*n* = 6)	NR (*n* = 11)	RL (*n* = 11)	rFFP (*n* = 11)
*Hemodynamics*				
Heart rate (bpm)	370 (344–385)	360 (348–372)	367 (340–377)	370 (344–380)
Saturation (%)	97 (95–99)	96 (95–98)	98 (97–99)	97 (94–98)
Total amount infused (mL)	0 (0–0)	0 (0–0)	2.79* (2.73–3.06)	2.85* (2.75–2.98)
*Blood count*				
Hb (mM)	10.1 (10.0–11.1)	11.4 (11.0–12.2)	10.6 (10.0–12.0)	11.7 (9.3–12.1)
Leukocytes (*10^9/L)	12.0 (7.1–14.6)	1.6* (1.4–2.4)	2.6* (1.1–8.6)	2.5* (1.6–7.5)
*Blood gas*				
Lactate (mM)	1.98 (1.62–2.05)	2.35* (2.11–2.62)	2.55* (2.37–2.72)	2.59* (2.09–2.92)
pH	7.42 (7.41–7.44)	7.44 (7.42–7.45)	7.43 (7.42–7.45)	7.43 (7.43–7.47)
pCO_2_ (mmHg)	45.1 (44.5–45.5)	48.0 (42.7–52.7)	47.2 (41.1–48.9)	50.2 (43.8–50.8)
BE (mM)	3.2 (2.1–4.6)	5.5* (4.2–7.2)	4.6 (3.6–5.9)	6.5* (5.6–6.9)
HCO3^−^ (mM)	28.2 (27.2–29.8)	31.2* (29.5–33.7)	30.2* (28.0–32.0)	31.6* (30.2–32.9)
Na + (mM)	138 (137–139)	138 (137–140)	139 (137–139)	139 (137–140)
K + (mM)	5.3 (5.1–5.4)	5.6* (5.4–5.8)	5.9* (5.3–6.6)	5.8* (5.6–6.2)
Ca2 + (mM)	1.21 (1.19–1.25)	1.13* (1.10–1.17)	1.12* (1.04–1.17)	1.10* (1.03–1.12)
Glucose (mM)	8.9 (7.5–12.2)	7.6 (7.1–9.3)	8.0 (7.4–8.9)	7.9 (6.8–8.2)

Data are presented as median (inter-quartile range). *p < 0.05 when compared to the sham group. *BE* base excess, *bpm* beats per minute, *Hb* haemoglobin, *NR* No resuscitation, *rFFP* rat fresh frozen plasma, *RL* Ringer’s lactate

**Table 2:** Parameters of systemic organ injury post resuscitation

Parameter	Sham (*n* = 6)	NR (*n* = 10)	RL (*n* = 11)	rFFP (*n* = 11)
Lactate (mM)	1.56 (0.87–1.69)	2.77* (1.96–3.51)	2.24* (2.10–2.71)	2.53* (2.13–2.71)
Hb (mM)	8.9 (8.3–9.4)	10.7* (10.3–11.1)	10.1* (9.4–11.6)	10.5* (9.7–11.3)
Glucose (mM)	10.3 (9.5–11.6)	7.4* (6.1–10.1)	8.2* (7.1–9.4)	7.3* (7.0–7.5)
ALT (U/L)	34.0 (30.8–38.3)	36.5 (30.8–39.5)	35.0 (30.0–38.3)	38.0 (23.0–40.0)
AST (U/L)	64.5 (59.8–73.5)	72.5 (56–78.8)	75.0 (54.0–82.5)	63.0 (56.0–103.5)
Albumin (g/L)	36.5 (33.5–39.0)	35.5 (33.3–39.5)	36.0 (34.0–38.0)	37.0 (38.0–35.0)
Creatinine (µmol/L)	23.5 (19.5–28.3)	23.0 (20.0–25.0)	23.0 (21.8–25)	23.0 (21.0–29.0)
Kidney W/D ratio	3.6 (3.4–4.0)	3.8 (3.6–3.9)	3.9* (3.7–4.1)	3.8 (3.7–3.9)

Data are presented as median (inter-quartile range). *p < 0.05 when compared to sham group. *ALT* alanine aminotransferase, *AST* aspartate aminotransferase, *Hb* Hemoglobin, *NR* No resuscitation, *rFFP* rat fresh frozen plasma, *RL* Ringer’s lactate, *W/D* Wet-to-dry

No differences were seen in pre-resuscitation parameters between the several groups with sepsis (Tables 1**, **Additional file 1: Table S2). During the experiment, 4 animals died. Two animals died during transfusion (both in hFFP group) and two animals were sacrificed upon reaching humane endpoints (1 after albumin and 1 non-resuscitated animal, Additional file 1: Results S1). All other animals concluded the study (*N* = 68). Of note, 5 animals of the hFFP group and 3 animals of the SDP group developed a transient increase of body temperature between 0.5 and 1 °C immediately after start of infusion.

### The effect of resuscitation fluids on pulmonary injury

Pulmonary vascular leakage was increased in septic animals compared to healthy controls (134 [105–165] vs 52 [33–112] µg/mL, *p* = 0.01, Fig. 1). Compared to the group resuscitated with Ringer’s Lactate, resuscitation with rFFP did not result in differences in any outcome of pulmonary vascular leakage (Fig. 1**)**, wet/dry ratios (Fig. 1), histology scores (Additional file 1: Table S1) and pulmonary bacterial outgrowth (Additional file 1: Fig.** S2**). Of interest, compared to non-resuscitated controls, animals resuscitated with rFFP had reduced pulmonary FITC-dextran vascular leakage (134 [105–165] vs 58 [44–89] µg/mL, *p* = 0.01, Fig. 1). No outcome parameter of pulmonary or endothelial injury differed between animals resuscitated with SDP, hFFP or 5% albumin (Additional file 1: Figs. S3, S4).

### The effect of resuscitation fluids on systemic injury

When compared to animals receiving no resuscitation, animals resuscitated with either Ringer’s lactate (*p* = 0.04) had increased kidney wet-to-dry ratios, whereas resuscitation with rFFP showed no difference (Table 2). However, when compared to resuscitation with RL, resuscitation with rFFP did not differ in terms of kidney edema. Also, no differences in levels of syndecan-1, TM, vWF and ICAM-1 were found (Fig. 2). No outcome parameter of systemic injury differed between animals resuscitated with SDP, hFFP or 5% albumin (Additional file 1: Fig. S4, Table S3).

## Discussion

This study investigated the effects of resuscitation with a fixed volume of fluids with either a low (crystalloid) or a high protein content (plasma), as well as the effects of different types of human-derived plasma products on endothelial and pulmonary injury in an experimental rat model of pneumosepsis.

This study did not show a benefit of resuscitation with rFFP on reducing endothelial permeability or organ edema when compared to resuscitation with crystalloids. This is in contrast to findings of experimental studies using models of hemorrhagic shock and sepsis, wherein plasma resuscitation compared to crystalloid resuscitation decreased endothelial permeability and improved outcome [17, 21, 22]. A potential explanation for a reduced effect in our study, is that in these previous studies, resuscitation volumes between groups differed, with two–threefold higher volumes of crystalloids than plasma, which could even reach 75 ml/kg [17, 21, 22]. In this study, a fixed volume was chosen for all groups to compare the effects of fluid content on endothelial and organ injury, independent of fluid volume. As our data do not suggest that administering protein rich fluids is unequivocally beneficial, we postulate that the beneficial effects of plasma found in previous experimental studies are largely related to administration of less volume. This would suggest that the amount and not the type of resuscitation fluid is the most important driver for aggravated endothelial injury. Multiple randomized controlled trials in sepsis showed that liberal use of crystalloids results in more kidney injury, respiratory failure and mortality compared to a restrictive approach [27–29]. On the other hand, a recent RCT found no advantage of a restrictive fluid resuscitation on mortality, although it should be noted that resuscitation volumes were quite low [30]. A systematic review and meta-analysis from the same authors report an overall very low quality of evidence with no benefit towards any strategy [31]. Therefore, whether administration of large volumes of crystalloids induce adverse outcome by worsening of endothelial function remains a topic of debate.

An alternative explanation for a limited protection from plasma is that the pneumosepsis model may have been too mild and did not cause a low albumin level. In line with this reasoning, albumin reduced 90-day mortality in septic shock patients, while this was not witnessed in patients with sepsis and no shock [32]. In addition, albumin may only benefit the subgroup of patients with hypo-albuminemia [14]. Therefore, protein-rich fluids such as plasma might only be favorable in the most severely ill septic shock patients and/or in those with low albumin levels. In the current model, even though septic animals all had bacteremia and showed signs of sepsis, most animals receiving no resuscitation and other types of supportive therapy survived 5 h. Thereby, it can be questioned whether resuscitation was indeed necessary. However, the presence of hyperlactatemia in the setting of sepsis reflects a clinical situation which would have triggered resuscitation [8].

An interesting finding is that even in this mild pneumosepsis model, animals receiving rat FFP had less lung leakage than non-resuscitated animals, and rat FFP did not aggravate kidney edema. This may suggest a beneficial effect on shock-induced permeability. However, this may be a chance finding and should be confirmed in follow-up studies.

We hypothesized that SDP plasma would be superior but this study found no difference between FFP and SDP on endothelial or organ injury. Conversely, SDP resuscitation increased kidney edema, while this was not witnessed in animals receiving FFP products. the use of cross-species transfusion products may have played a role, as human plasma products induced a transient increase of body temperature in some animals and 2 animals in the hFFP group unexpectedly died during transfusion.

This study has several limitations. First, as already mentioned, hemodynamic monitoring was absent. Second, due to higher data variability than expected, the used sample size may have been too low to show potential differences between interventions. In addition, we transfused human-derived plasma products to rats possibly resulting in cross-species reactions which could skew advantageous effects. We chose human products as it enabled us to compare the effects between SDP and FFP products. Multiple control groups were added to the model to control for this. However, this does increase the probability of type 1 errors. Rats were followed for 5 h after start of resuscitation. Therefore, long term effects on endothelial and organ injury are missed. Only male rats were used in the current model reducing the translational generalizability of the found results. This choice was made to limit the amount of animals used. Female rats develop less endothelial injury due to estrogen production. Therefore, including female rats would reduce the effect size warranting a larger sample size. Finally, no antibiotics, mechanical ventilation or vasopressors were used in the current model, as we aimed for a model that reflects primary resuscitation upon hospital admittance. Obviously, this choice reduces translational value to patients whom already received other organ support, such as mechanical ventilator. However, this was chosen deliberately as we aimed to investigate the direct effects of the used resuscitation products on the endothelium reducing possible confounders.

## Conclusion

In conclusion, resuscitation rFFP when compared to an equal resuscitation volume of crystalloids did not reduce markers of glycocalyx degradation, endothelial injury and organ injury, but may limit shock-induced pulmonary leakage. In addition, different types of human-derived plasma products including 5% human albumin, FFP and SDP resulted in similar endothelial and pulmonary injury.

### Supplementary Information

**Additional file 1: **Supplementary data.

**Additional file 2. **ARRIVE guidelines.

## Data Availability

The data sets generated and/or analysed during the current study are not publicly available but are available from the corresponding author on reasonable request.

## Abbreviations

ALT: Alenine transaminase
AST: Aspartate aminotransferase
Bpm: Beats per minute
CFU: Colony forming units
COS: Colombian Agar with 5% sheep blood plates
Hb: Hemoglobin
FITC: Fluorescein isothiocyanate
hFFP: Human-derived fresh frozen plasma
H&E: Hematoxylin and Eosin
ICAM-1: Intercellular adhesion molecule 1
IQR: Interquartile range
NR: No resuscitation
rFFP: Rat-derived fresh frozen plasma
RIPA: Radio immunoprecipitation assay buffer
RL: Ringer’s Lactate
SDP: Solvent detergent plasma
TM: Thrombomodulin
vWF: Von Willebrand factor
W/D ratio: Wet dry ratio
