# Supplementary material for: The effects of resuscitation with different plasma products on endothelial permeability and organ injury in a rat pneumosepsis model
Source: Intensive Care Med Exp. 2023 Sep 20;11:62. doi: 10.1186/s40635-023-00549-9 (PMC10511387; doi:10.1186/s40635-023-00549-9)
Supplement: Supplementary file 1 — Additional file 1: Supplementary data. [file 40635_2023_549_MOESM1_ESM.docx]

**Additional file 1:** Supplementary data

- **Table S1:** Lung injury assessment scoring list:
- **Figure S1:** Schematic overview of experimental model
- **Figure S2:** Bacterial outgrowth in lung homogenate
- **Figure S3:** Markers of pulmonary injury post resuscitation of animals receiving human plasma products (T=5)
- **Figure S4:** Markers of endothelial injury post resuscitation of animals receiving human plasma products (T=5)
- **Table S2:** Pre-resuscitation parameters of animals receiving human plasma products (T=0)
- **Table S3:** Parameters of systemic organ injury post resuscitation of animals receiving human plasma products (T=5)
- **Results S1:** Mortality and humane endpoints:
- **Results S2:** Pneumosepsis model Monitoring sheet.

**Table S1:** Lung injury assessment scoring list

| **Group:** | **Sham** | **NR** | **RL** | **Albu 5%** | **rFFP** | **hFFP** | **SDP** |
| --- | --- | --- | --- | --- | --- | --- | --- |
| **Interstitial inflammation** | 0  (0-1) | 3*  (1-3) | 2*  (1-2) | 2  (1-2) | 2*  (2-3) | 2*  (1-2) | 2*  (1-3) |
| **Endothelialitis** | 0  (0-0) | 0  (0-1) | 1*  (0-2) | 0  (0-0) | 0  (0-1) | 0  (0-1) | 1*  (0-2) |
| **Bronchitis** | 0  (0-0) | 2*  (1-3.5) | 3*  (1-3) | 3*  (2-3) | 2*  (2-3) | 2*  (2-3) | 3*  (2-3) |
| **Edema** | 0  (0-1) | 2*  (2-4) | 3*  (1-3) | 3*  (3-3) | 3*  (2-3) | 3*  (2-3) | 3*  (2-3) |
| **Thrombi formation** | 0  (0-0) | 0  (0-0) | 0  (0-0) | 0  (0-0) | 0  (0-0) | 0  (0-0) | 0  (0-0) |

Severity was assessed based on a scale from 0-4 (0, absent; 1, mild; 2, moderate; 3, severe; 4 = very severe). * = P < 0.05 when compared to the sham group, Albu = Albumine 5%, hFFP = human fresh frozen plasma, NR= No resuscitation, rFFP = rat fresh frozen plasma, RL = Ringer’s lactate, SDP = solvent detergent plasma.

**Figure S1:** Schematic overview of experimental model


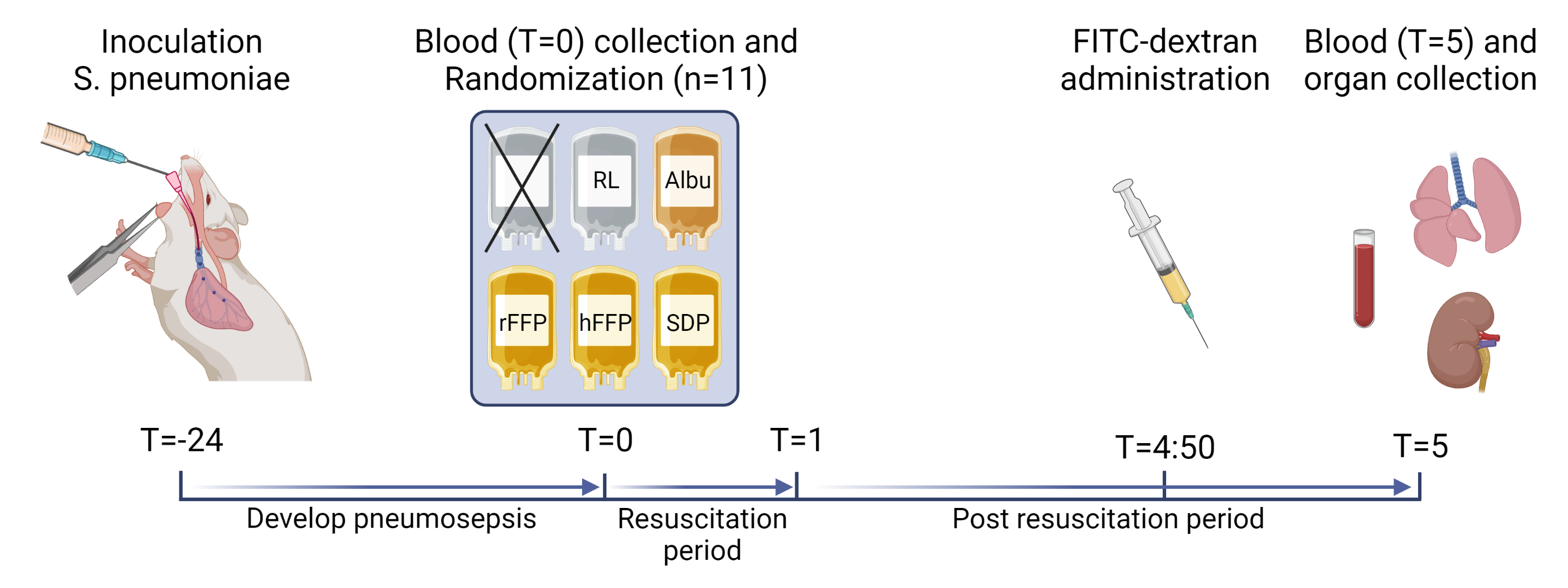


Description of pneumosepsis rat model. Animals were intratracheally inoculated with sham inoculate or 3-5 x 10^8^ *Streptococcus pneumonia*e whereafter they developed pneumosepsis. 24 hours after inoculation, animals were anesthetized using isoflurane and blood was collected (T0). Thereafter, animals were randomized to no resuscitation or resuscitation with Ringer’s lactate (RL), Albumine 5% (Albu), rat derived fresh frozen plasma (rFFP), human derived fresh frozen plasma (hFFP) or solvent detergent plasma (SDP). Fluids were administered at 8 ml/kg for 1 hour after which resuscitation was stopped and animals were allowed to wake up. Four hours and forty-five minutes after start of transfusion, animals were anesthetized using isoflurane and FITC-labelled 70 kDa dextran was administered. This label circulated for 10 minutes. Then, blood was collected by heart puncture, quickly followed by a bolus of heparin, tying-off of the hilum of left lung and left kidney (for later W/D ratio assessments). The circulation was flushed using 50ml of 0.9% NaCl against gravitational force. Organs were harvested for endothelial leakage assessments and organ failure scores.

**Figure S2:** Bacterial outgrowth in lung homogenate


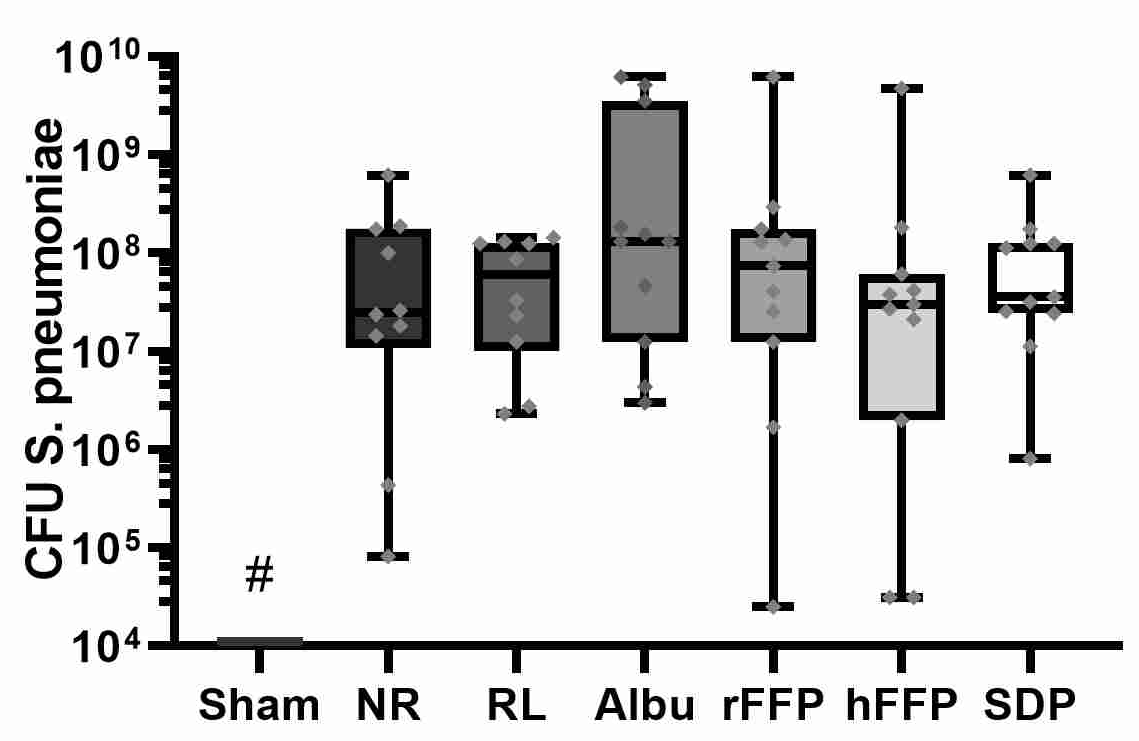


Data are presented as boxplots with median, interquartile ranges and minimum and maximum values showing all individual data points

**Figure S3:** Markers of pulmonary injury post resuscitation of animals receiving human plasma products (T=5)


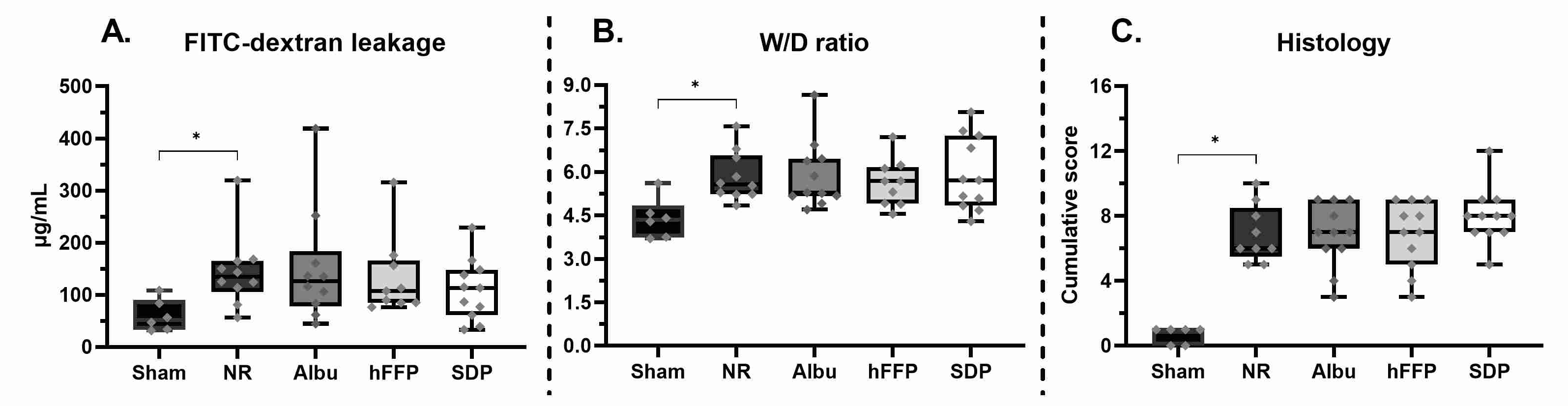


Data are presented as boxplots with median, interquartile ranges and minimum and maximum values showing all individual data points. *p < 0.05. Albu = 5% Albumin, FITC = Fluorescein isothiocyanate, hFFP = human fresh frozen plasma, NR = no resuscitation, W/D = Wet dry, SDP = solvent detergent plasma.

**Figure S4:** Markers of endothelial injury post resuscitation of animals receiving human plasma products (T=5)


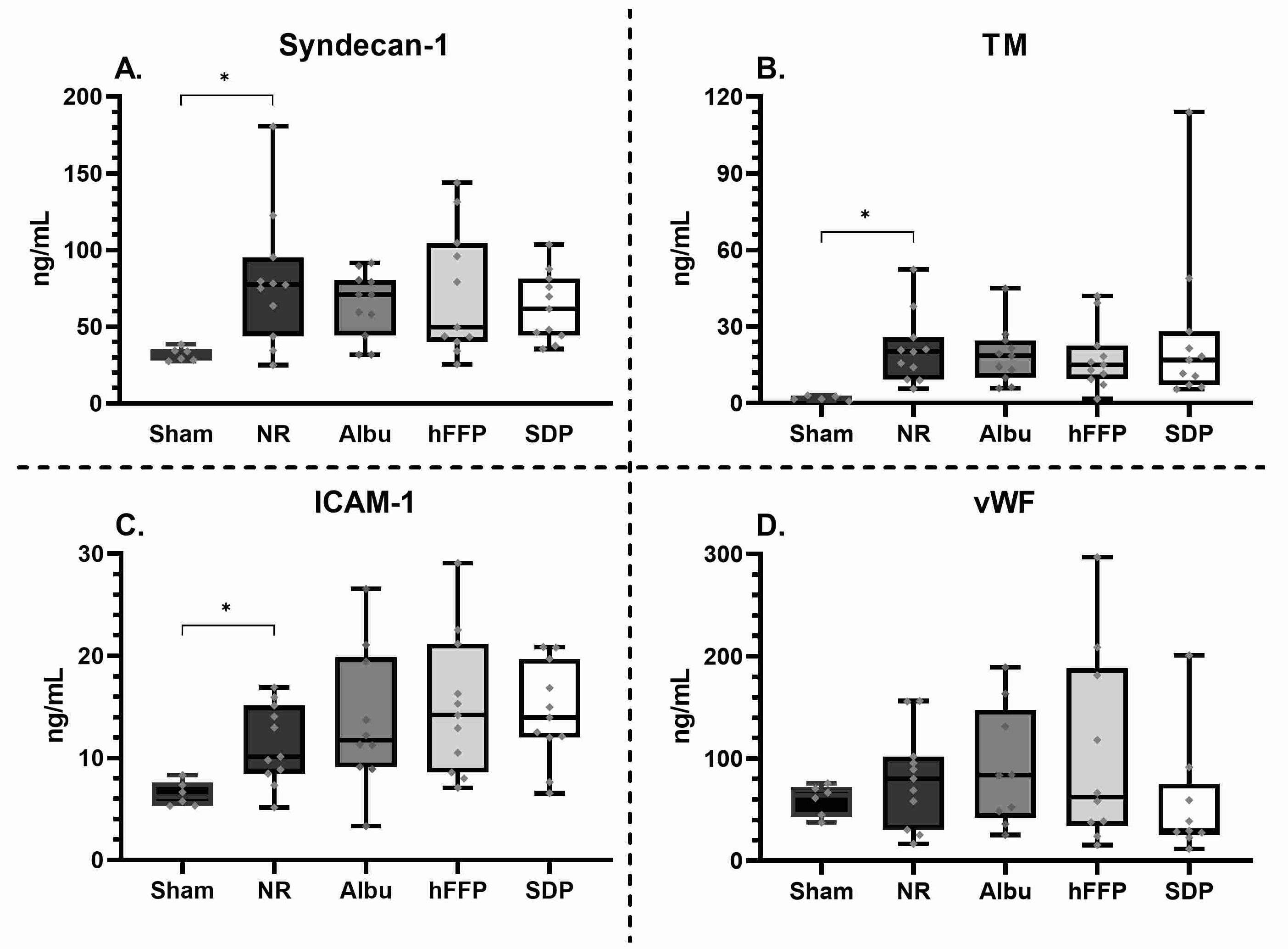


Data are presented as boxplots with median, interquartile ranges and minimum and maximum values showing all individual data points. *p < 0.05. Albu = 5% Albumin, hFFP = human fresh frozen plasma, ICAM-1 = intercellular adhesion molecule 1, NR = no resuscitation, SDP = solvent detergent plasma, TM= Thrombomodulin, vWF = von Willebrand factor.

**Table S2:** Pre-resuscitation parameters of animals receiving human plasma products (T=0)

| **Parameter** | **Sham (n=6)** | **NR (n=11)** | **Albu 5% (n=11)** | **hFFP (n=11)** | **SDP**  **(n=11)** |
| --- | --- | --- | --- | --- | --- |
| *Hemodynamics* | | | | | |
| **Heart rate**  **(bpm)** | 370  (344-385) | 360  (348-372) | 365  (338-381) | 355  (319-373) | 341  (323-371) |
| **Saturation**  **(%)** | 97  (95-99) | 96  (95-98) | 95  (93-98) | 96  (95-98) | 98  (95-98) |
| **Total amount infused (mL)** | 0  (0-0) | 0  (0-0) | 2.95*  (2.68-3.13) | 2.97*  (2.84-3.06) | 2.90*  (2.70-2.98) |
| *Blood count* | | | | | |
| **Hb**  **(mM)** | 10.1  (10.0-11.1) | 11.4  (11.0-12.2) | 11.1  (10.8-11.7) | 8.8  (7.9-11.9) | 11.5  (10.6-11.7) |
| **Leukocytes**  **(*10^9/L)** | 12.0  (7.1-14.6) | 1.6*  (1.4-2.4) | 4.1*  (1.5-9.1) | 2.0*  (0.8-6.9) | 3.2*  (2.4-9.1) |
| *Blood gas* | | | | | |
| **Lactate (mmol/L)** | 1.98  (1.62-2.05) | 2.35*  (2.11-2.62) | 2.51*  (2.41-3.04) | 2.23*  (1.92-2.79) | 2.65*  (2.35-2.93) |
| **pH** | 7.42  (7.41-7.44) | 7.44  (7.42-7.45) | 7.41  (7.38-7.44) | 7.45  (7.40-7.46) | 7.43  (7.37-7.46) |
| **pCO_2_**  **(mmHg)** | 45.1  (44.5-45.5) | 48.0  (42.7-52.7)) | 49.6  (44.3-54.2) | 47.9  (47.1-54.1) | 46.4  (41.4-55.5) |
| **BE**  **(mM)** | 3.2  (2.1-4.6) | 5.5*  (4.2-7.2) | 4.6  (2.6-6.9) | 6.2*  (4.4-6.9) | 5.2  (1.4-7.4) |
| **HCO3^-^**  **(mM)** | 28.2  (27.2-29.8) | 31.2*  (29.5-33.7) | 31*  (28.6-32.2) | 32*  (30.1-32.9) | 30.3  (26.5-33.7) |
| **Na+**  **(mM)** | 138  (137-139) | 138  (137-140) | 139  (138-140) | 140  (139-141) | 138  (136-138) |
| **K+**  **(mM)** | 5.3  (5.1-5.4) | 5.6*  (5.4-5.8) | 5.6*  (5.4-5.8) | 5.4*  (5.3-7.6) | 5.7*  (5.4-5.9) |
| **Ca2+**  **(mM)** | 1.21  (1.19-1.25) | 1.13*  (1.10-1.17) | 1.16*  (1.09-1.19) | 1.10*  (1.02-1.15) | 1.11*  (1.05-1.15) |
| **Glucose**  **(mM)** | 8.9  (7.5-12.2) | 7.6  (7.1-9.3) | 7.6  (7.4-9.5) | 7.2  (6.8-8.0) | 7.3  (6.7-8.7) |

Data are presented as median (inter-quartile range). * p < 0.05 when compared to the sham group. Albu = Albumin, BE = base excess, bpm = beats per minute, Hb = haemoglobin, hFFP = human fresh frozen plasma, NR= No resuscitation, SDP = solvent detergent plasma

**Table S3:** Parameters of systemic organ injury post resuscitation of animals receiving human plasma products (T=5)

| **Parameter** | **Sham (n=6)** | **NR (n=10)** | **Albu 5% (n=10)** | **hFFP (n=9)** | **SDP (n=11)** |
| --- | --- | --- | --- | --- | --- |
| **Lactate**  **(mmol/L)** | 1.56  (0.87-1.69) | 2.77*  (1.96-3.51) | 2.12*  (2.04-2.67) | 2.58*  (1.95-2.81) | 2.66*  (2.51-3.09) |
| **Hb**  **(mM)** | 8.9  (8.3-9.4) | 10.7*  (10.3-11.1) | 10.4*  (9.6-10.9) | 10.1*  (9.1-11.7) | 10.3*  (9.3-11.0) |
| **Glucose**  **(mM)** | 10.3  (9.5-11.6) | 7.4*  (6.1-10.1) | 7.9*  (7.6-8.1) | 7.6*  (7.1-8.9) | 7.2*  (5.7-7.7) |
| **ALT**  **(U/L)** | 34.0  (30.8-38.3) | 36.5  (30.8-39.5) | 34.5  (29.8-37.3) | 33.0  (29.0-35.0) | 36.0  (34.0-43.0) |
| **AST**  **(U/L)** | 64.5  (59.8-73.5) | 72.5  (56-78.8) | 86.0  (62.0-95.5) | 64.5  (51.5-80.3) | 92.0  (47.8-108.8) |
| **Albumin**  **(g/L)** | 36.5  (33.5-39) | 35.5  (33.3-39.5) | 37  (35.5-38.3) | 37.0  (36.0-38.0) | 37.5  (36.0-39.3) |
| **Creatinine**  **(µmol/L)** | 23.5  (19.5-28.3) | 23.0  (20.0-25.0) | 21.0  (20.0-25.0) | 22.0  (20.0-24.0) | 22.5  (20.8-24.0) |
| **Kidney W/D ratio** | 3.6  (3.4-4.0) | 3.8  (3.6-3.9) | 4.0*  (3.7-4.1) | 3.8  (3.7-3.9) | 3.9*  (3.8-4.1) |

Data are presented as median (inter-quartile range). * p < 0.05 when compared to sham group. ALT = alanine aminotransferase, AST: aspartate aminotransferase, bpm = beats per minute, Hb = Hemoglobin, CFU = colony forming units, hFFP = human fresh frozen plasma, NR= No resuscitation, SDP = solvent detergent plasma

**Results S1:** Mortality and humane endpoints

- 2 rats receiving hFFP died during transfusion as they stopped breathing.
- 1 rat receiving albumin was sacrificed 4 hours after receiving intervention upon showing severe dyspnea with abdominal breathing which was defined as a humane end point.
- 1 rat receiving Ringers Lactate was sacrificed immediately after receiving infusion upon showing severe dyspnea with abdominal breathing which was defined as a humane end point.

**Results S2:** Pneumosepsis model Monitoring Sheet

**Animal #:** **Date:**

Fur aspect Actively grooming Dulling of hair coat Rough hair coat Piloerection

Activity Normal Reduced activity disturbed No activity disturbed Nil activity disturbed or
 Reduced activity stimulated stimulated

Behavior Normal, no Slightly hunched, Hunched with stiff Hunched with no
 abd splinting moving freely, mild movement/posture, movement stimulated,
 splinting moderate splinting severe splinting

Face Normal Normal eyelid opening Orbital tightening, Eyelids closed,
 when disturbed moderate grimacing obvious grimacing

Diarrhea None Mild Moderate Severe

Respiratory None Mild dyspnea Moderate dyspnea Severe dyspnea with
distress abdominal breathing

Score 1 2 3 4

A score of 4 in any of the categories results in early termination.

Adapted from:

- Huet O, Ramsey D, Miljavec S, Jenney A, Aubron C, Aprico A, Stefanovic N,Balkau B, Head G, de Haan J, et al.: Ensuring animal welfare while meeting scientific aims using a murine pneumonis model of septic shock. Shock 39(6):488-494, 2013
- Chang R, Holcomb JB, Johansson PI, Pati S, Schreiber MA, Wade CE. Plasma Resuscitation Improved Survival in a Cecal Ligation and Puncture Rat Model of Sepsis. Shock. 2018 Jan;49(1):53-61. doi: 10.1097/SHK.0000000000000918. PMID: 28591008; PMCID: PMC5718978.
